# A comprehensive analysis of Usutu virus (USUV) genomes revealed lineage-specific codon usage patterns and host adaptations

**DOI:** 10.3389/fmicb.2022.967999

**Published:** 2023-01-12

**Authors:** Jianglin Zhou, Yaling Xing, Zhe Zhou, Shengqi Wang

**Affiliations:** Beijing Institute of Microbiology and Epidemiology, Beijing, China

**Keywords:** codon usage, natural selection, mutation pressure, evolution, Usutu virus

## Abstract

The Usutu virus (USUV) is an emerging arbovirus virus maintained in the environment of Afro-Eurasia *via* a bird-mosquito-bird enzootic cycle and sporadically infected other vertebrates. Despite primarily asymptomatic or mild symptoms, humans infected by USUV can develop severe neurological diseases such as meningoencephalitis. However, no detailed study has yet been conducted to investigate its evolution from the perspective of codon usage patterns. Codon usage choice of viruses reflects the genetic variations that enable them to reconcile their viability and fitness toward the external environment and new hosts. This study performed a comprehensive evolution and codon usage analysis of USUVs. Our reconstructed phylogenetic tree confirmed that the circulation viruses belong to eight distinct lineages, reaffirmed by principal component analysis based on codon usage patterns. We also found a relatively small codon usage bias and that natural selection, mutation pressure, dinucleotide abundance, and evolutionary processes collectively shaped the codon usage of the USUV, with natural selection predominating over the others. Additionally, a complex interaction of codon usage between the USUV and its host was observed. This process could have enabled USUV to adapt to various hosts and vectors, including humans. Therefore, the USUV may possess a potential risk of cross-species transmission and subsequent outbreaks. In this respect, further epidemiologic surveys, diversity monitoring, and pathogenetic research are warranted.

## Introduction

Usutu virus (USUV) is an emerging arbovirus belonging to the genus *Flavivirus* in the family *Flaviviridae*. USUV is a member of the Japanese encephalitis virus (JEV) serocomplex, genetically close to human pathogens JEV, West Nile virus (WNV), and Murray Valley encephalitis virus (MVEV; Roesch et al., 2019). Like other flaviviruses, USUV has a + ssRNA genome comprised of 11,064 nucleotides that encodes one open reading frame (ORF) and two flanking untranslated regions (Bakonyi et al., 2004; Pierson and Diamond, 2020). The ORF that encodes a polyprotein of 3,434 amino acids will be enzymatically cleaved into three structural proteins (C, prM, E) and seven non-structural proteins (NS1, NS2A, NS2B, NS3, NS4A, NS4B, and NS5). Since first isolated in 1959 from a *Culex neavei* mosquito in Swaziland, USUV has continuously circulated within Africa and later spread across Europe (Vilibic-Cavlek et al., 2020). USUV is sustained in an enzootic cycle among wild birds as amplifying hosts (primarily in *Turdus merula*) and mosquitoes as vectors (mainly in *Cx. pipiens*). Humans and other mammals, including rodents, horses, bats, and deer, are sporadically infected and considered to be dead-end hosts (Roesch et al., 2019; Vilibic-Cavlek et al., 2020). To date, at least 100 cases of acute infection have been described in humans, with symptoms ranging from mild or asymptomatic to severe neurological disease (Clé et al., 2021). Besides epidemic potential, USUV may also represent a risk for blood safety, especially in the context of co-circulation with WNV and probably underestimated circulation of USUV (Domanović et al., 2019; Llorente et al., 2019). Therefore, an exhaustive study of the replication and evolution of USUVs is warranted.

The redundancy of genetic code allows organisms to regulate their efficiency and accuracy of protein production while preserving the same amino-acid sequences (Stoletzki and Eyre-Walker, 2007; Nasrullah et al., 2015). During protein translation in a certain species or cell, some codons are used more frequently than others, a phenomenon known as codon usage bias (CUB; Behura and Severson, 2013; Li et al., 2018). Previous studies indicated that CUB is common in three domains and viruses and is influenced by many factors, such as mutation pressure, natural or translation selection, dinucleotide abundance, and external environment (Moratorio et al., 2013; Nasrullah et al., 2015; Butt et al., 2016; Luo et al., 2020). Considering the entire parasitism of viruses, it depends on host tRNA to produce viral proteins, which create a translational selection that leads to the assimilation of viral CUB to its host CUB. What’s more, the virus tended to be more similar to that of symptomatic hosts than that of natural hosts (Chen et al., 2020). Hence, the interactions between the virus and its host are expected to influence viral viability, fitness, evolution, and evasion of the host’s immune responses (Nasrullah et al., 2015; Butt et al., 2016). Studying CUB thus supplies a novel perspective on virus evolution and can deepen our understanding of the biological properties of USUVs and aid in potential vaccine design. However, to our knowledge, there is only one report on the codon adaptation index for just four hosts within a fraction of USUVs (Zecchin et al., 2021); no detailed analysis of codon usage of USUVs has been published.

In this study, we comprehensively analyzed the phylogenetic relationships and codon usage patterns of USUVs. We also explored the possible key factors responsible for the CUB of USUV as well as its adaptation to various hosts. Our results show a novel perspective regarding molecular evolution in USUV.

## Materials and methods

### Dataset retrieval and annotation

All the whole genomes of USUV were collected from the GenBank database on March 10, 2022. These genomes were clustered by CD-HIT (cd-hit-est) at 100% identity and only one sequence was kept for every cluster to remove redundancy (Fu et al., 2012). The USUV genomes were annotated by VADR (Schäffer et al., 2020). Genomes whose ORF has fuzzy coordinates or non-(A, C, G, U) nucleotides were removed. Finally, a total of 368 genomes were analyzed in this study. Detailed genomes are listed in Supplementary Table S1.

### Recombination and phylogenetic analysis

Potential recombination events in USUV coding sequences were detected by the Genetic Algorithm for Recombination Detection (GARD) using the Datamonkey web service (Kosakovsky Pond et al., 2006; Weaver et al., 2018). All genome sequences were aligned by MAFFT (Katoh and Standley, 2013). The maximum likelihood (ML) phylogenetic tree was constructed by IQ-TREE with 1,000 replications of ultrafast bootstrap resampling (Hoang et al., 2018) and SH-aLRT test (Minh et al., 2020). The model GTR + F + I + G4 was selected using the built-in ModelFinder (Kalyaanamoorthy et al., 2017). The tree was visualized using the ggtree package (Yu et al., 2017).

### Nucleotide and codon composition analysis

The frequencies of A, U, G, and C, overall GC content, GC percentage at the first (GC1s), second (GC2s), third (GC3s) codon position, and the average of GC1s and GC2s (GC12s) were calculated by seqinr package (Charif and Lobry, 2007b). The frequencies of A, U, G, and C at the third positions in the synonymous codons (A3s, U3s, G3s, C3s) were calculated by CodonW.^1^ Five codons without synonymous codons, including AUG, UGG, UAG, UAA, and UAG, were excluded from this analysis.

### Relative synonymous codon usage (RSCU) analysis

The RSCU values represent the usage frequencies of synonymous codons in protein excluding the effect of the sequence length and amino acid compositions (Sharp and Li, 1986). The RSCU value was estimated using the seqinr package as follows:


$$RSCU=\frac{X_{ij}}{\frac{1}{N_{i}}\sum_{j}^{N_{i}}X_{ij}}$$

Where *X*_*ij*_ is the observed number of the *j*th codon for the *i*th amino acid, which has *N*_*i*_ kinds of alternative synonymous codons. Codons with RSCU values >1.6 are considered as over-represented, whereas <0.6 reflected under-represented ones.

### Principal component analysis (PCA)

PCA is widely used to resolve the relationship between the multivariate and samples. Here, each ORF represented by a 59-dimensional vector was transformed into several principal components (PCs). The PCA analysis was performed using the factoextra package (Kassambara and Mundt, 2017).

### Relative dinucleotide abundance analysis

The relative abundance of sixteen dinucleotides in USUV coding sequences was calculated using the previously described formula as shown below (Kariin and Burge, 1995):


$$rho_{xy}=\frac{f_{xy}}{f_{x}\cdot f_{y}}$$

Where the *f_x_* and *f_y_* denote the observed frequency of mononucleotide X and Y, respectively; and *f_xy_* represents the observed frequency of dinucleotide XY. The expected *rho_xy_* = 1 means the dinucleotide randomly occurred. Generally, for *rho_xy_* > 1.23 or < 0.78, the dinucleotide XY is considered to be over-represented or under-represented. The *rho* values were estimated by the seqinr package (Charif and Lobry, 2007a).

### The effective number of codons (ENC) estimation

The ENC value indicates the extent of CUB, ranging from 20 to 61 (Comeron and Aguadé, 1998). The smaller ENC value represents a stronger CUB. The ENC values were estimated by the seqinr package.

### ENC-plot analysis

To identify factors influencing CUB, ENC-plot analysis was performed by plotting the ENC values against the GC3s. Genes whose codon usage is only constrained by mutation pressure will locate on or around the expected curve. Otherwise, natural selection exerts a more powerful influence. The expected ENC value was inferred using the below formula:


$$ENC_{expected}=2+s+\frac{29}{s^{2}+{\left(1-s\right)}^{2}}$$

Where the *s* represents values of GC3s (Wright, 1990).

### Neutrality plot analysis

The neutrality plot was used to determine the dominant factors (mutation pressure or natural selection) influencing CUB (Sueoka, 1988). The GC12s values (y-axis) were plotted against the GC3s values (x-axis). Mutation pressure is considered the dominant force shaping codon usage if the coefficient of GC3s is statistically significant and close to 1. The slope value is closer to 0 means a higher influence from natural selection.

### Codon adaption index (CAI) calculation

The CAI analysis is a quantitative method that is applied to evaluate the adaptiveness of a gene toward the codons of highly expressed genes (Sharp and Li, 1987). CAI values of USUV coding sequences were calculated using the local version of CAIcal (Puigbò et al., 2008), to the codon usage patterns of its hosts and vectors. A total of 14 species representing four categories of hosts and vectors, including birds (*T. merula*, *Sturnus vulgaris*, *Passer domesticus*, *Alauda arvensis*, and *Bubo scandiacus*), mosquitoes (*Cx. pipiens* pallens, *Cx. quinquefasciatus*, *Aedes albopictus*, and *Ae. aegypti*), human (*Homo sapiens*) and non-human mammals (NHM, *Pipistrellus pipistrellus*, *Rattus rattus*, *Capreolus capreolus*, and *Equus caballus*), were obtained from the Codon and Codon-Pair Usage Tables (CoCoPUTs) database on March 21, 2022 (Alexaki et al., 2019). The CAI value by reference codon usage pattern ranges from 0 to 1 with higher CAI values signifying better viral adaptation to the corresponding host.

### Relative codon deoptimization index (RCDI) calculation

The RCDI value measures the deoptimization of the USUV toward that of its hosts. An RCDI value of 1 indicates that the virus pursues the codon usage pattern of the host and exhibits a host-adapted codon usage preference. Contrarily, an RCDI value >1 indicates the codon usage pattern of the virus deviates from its host. The RCDI values were calculated for the 14 species using CAIcal (Puigbò et al., 2008).

### Similarity index analysis

The similarity index [SiD or D(A, B)] is an indicator to estimate the overall effect of host codon usage on viral codon usage. The SiD values of USUV for fourteen hosts were calculated using the following formula (Zhou et al., 2013):


$$R\left(A,B\right)=\frac{\sum_{i=1}^{59}a_{i}\cdot b_{i}}{\sqrt{\sum_{i=1}^{59}a_{i}{}^{2}\cdot \sum_{i=1}^{59}b_{i}{}^{2}}}$$

$$D\left(A,B\right)=\frac{1-R\left(A,B\right)}{2}$$

Where a*_i_* and b*_i_* represents the RSCU value of 59 synonymous codons for the USUV and the host, respectively. D(A, B) indicates the potential effect of the overall codon usage of the host on that of USUV, ranging from 0 to 1. A Higher SiD value means a greater impact from the host on USUV codon usage.

### Correlation and statistics analysis

Spearman’s rank correlation analysis was performed to determine the relationships among the genomic composition, ENC, Aromo, Gravy, and the first two axes of PCA. A two-sided Dunn’s test was used to the statistical significance between groups. *p* values were corrected using Benjamini-Hochberg (BH) procedure and 0.05 was used as the significance threshold.

## Results

### Phylogenetic analysis of USUV

**Figure 1:**
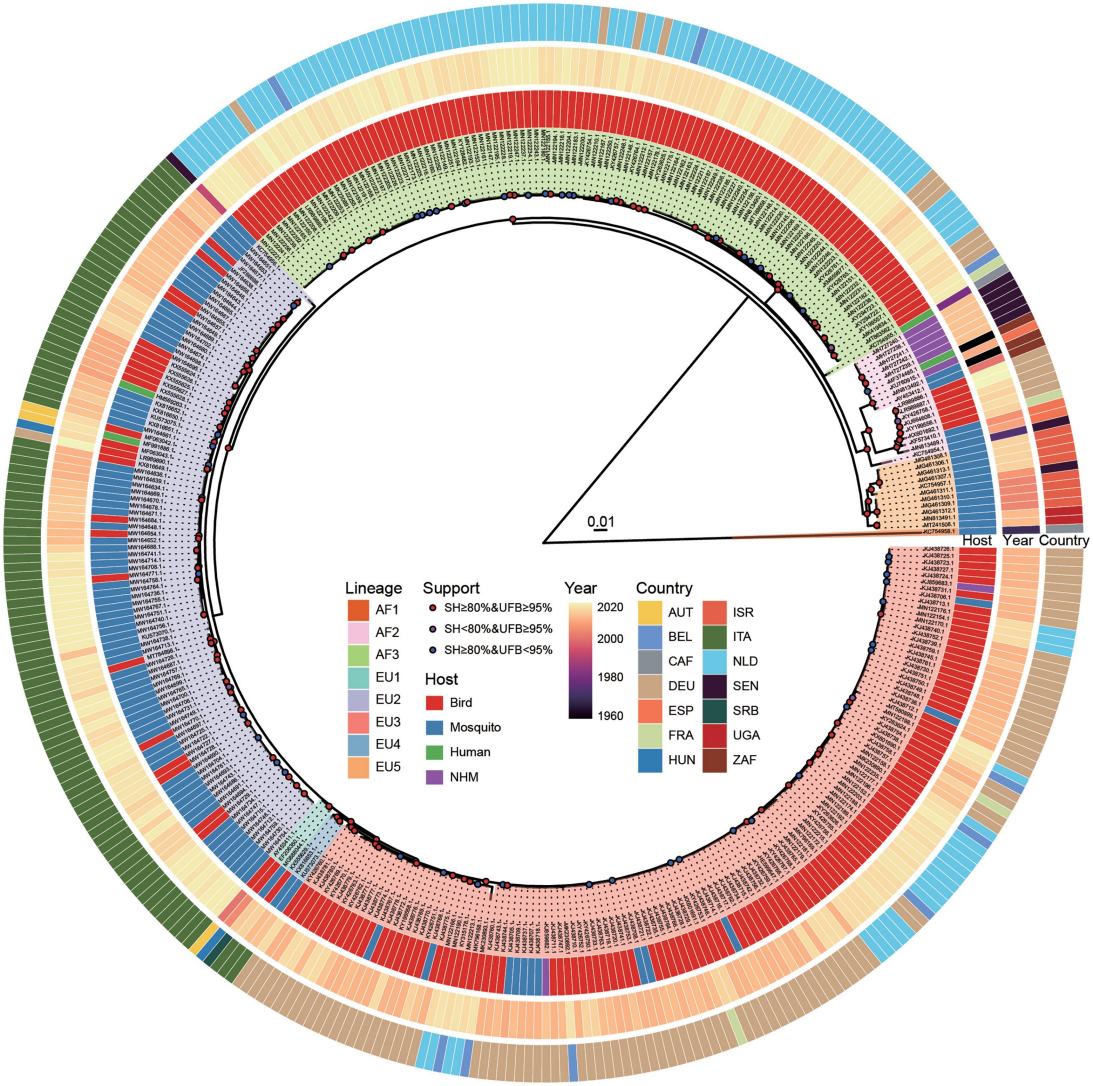
Phylogenetic tree of 368 whole genomes of Usutu virus (USUV) based on IQ-TREE. Branch supports were displayed in colored points according to the combination of 1,000 replicates of ultrafast bootstrap (UFB) and SH-aLRT test (SH). The background of the USUV strain labels was filled based on lineage classification. The circular color blocks at the periphery of the tree are distributed from inside to outside indicating the isolation host, collection year, and the country of the isolates, respectively.

GARD analysis found no evidence of recombination events among the 368 USUV strains, hence all of them were included for subsequent phylogenetic and codon usage analyses. The obtained ML phylogeny shows that these USUV strains fell into eight distinct African (AF) and European (EU) lineages, namely AF1-3, and EU1-5 (Figure 1). The AF1, which contains only one strain from an African mosquito, is distantly far away from the others. Except for the EU4, which only includes viruses from Italy, AF2, AF3, EU1, EU2, EU3 and EU5 are widespread in varied hosts of many countries and continents.

### G and A nucleotides are more abundant in the USUV coding sequences

The nucleotide composition was analyzed to evaluate its potential impact on codon usage of USUV. Here we found that the most frequent mononucleotide was G, with a mean ± standard deviation (SD) value of 28.34 ± 0.08%, followed by A (27.03 ± 0.08%), C (22.87 ± 0.07%), and U (21.76 ± 0.08%). The C3s, A3s, G3s, and U3s was 34.07 ± 0.22%, 30.86 ± 0.28%, 30.71 ± 0.26%, and 26.48 ± 0.20%, respectively. The overall GC content (51.21 ± 0.06%) was slightly higher than that of AU. The GC1s (56.91 ± 0.09%) and GC3s (52.34 ± 0.17%) values were higher than GC2s (44.91 ± 0.05%) and GC12s (50.91 ± 0.06%). The detailed nucleotide compositions of strains are listed in Supplementary Table S2. Therefore, although the USUV coding sequences were GC-rich, mononucleotides G and A were more abundant. Significant differences (adjusted *p* < 0.05) were also noticed in the average GC, GC1s, GC2s, and GC3s values of USUV strain in various lineages and hosts (Supplementary Figures S1A,B).

Furthermore, nucleotide composition was estimated on every specific gene of USUV. The mononucleotide G or A were more abundant in all genes, except for the *NS4B* (C/G, i.e., most frequent mononucleotide is C, followed by G). Although the mean values of G3s and A3s were highest in some genes (*NS2A* and *NS4A*, *NS2B,* and *NS4B*, respectively), mean C3s was highest in most genes, which was consistent with that of the whole coding sequences. The GC content was slightly higher than AU in every gene except for the *prM* (0.49 ± 0.003) and *NS2B* (0.49 ± 0.009). Meanwhile, the mean GC3s were higher than AU3s in all genes except for the *NS2B* (0.40 ± 0.02) and *NS4B* (0.49 ± 0.01; Supplementary Figure S1C). Thus, despite the anomalies of a few genes, the nucleotide composition of most genes follows a similar trend as that of the whole coding sequence. Taken together, these results confirmed that nucleotide compositions of the USUV viruses are complicated and imbalanced, implying a biased codon usage.

### CUB among the USUV

**Figure 2:**
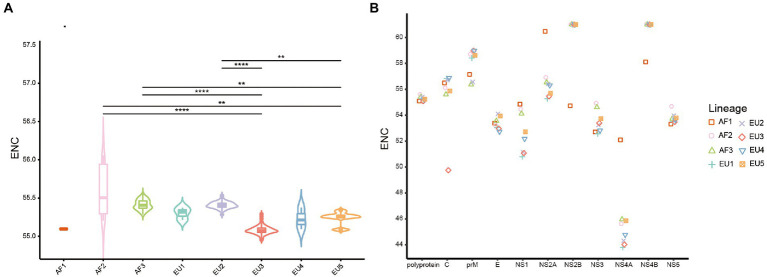
The effective number of codons (ENC) values distribution. **(A)** The violin plots with inner boxplots showed the ENC values of polyproteins of USUV in different lineages. Horizontal lines of inner boxplots represent median values, with whiskers extending to the farthest data point within a maximum of 1.5 × interquartile range. Benjamini-Hochberg (BH)-corrected Dunn’s test was performed to infer the significance between lineages. All differences with *p* < 0.05 are indicated. **p < 0.01; ***p < 0.001; ***p < 0.0001. **(B)** The scatter plot shows the mean ENC values of individual coding sequences of USUV, considering the lineage classification of strains. The lineages are indicated by distinct scatter shapes and colors.

The ENC values were calculated to estimate the degree of USUV CUB. The ENC values of whole coding sequences ranged from 54.95 to 56.05 (mean 55.30 ± 0.19), irrespective of lineage (Supplementary Table S2). Concerning the lineage classification of complete coding sequences, a significantly highest ENC value of 55.60 ± 0.33 was observed in the AF2 lineage while the lowest ENC value of 55.08 ± 0.05 was observed in the EU3 lineage (*p* < 0.0001; Figure 2A). Analyzing individual genes showed that *NS4A* had the lowest ENC value (44.82 ± 1.25), while *NS2B* and *NS4B* had the comparable highest ENC value of 60.98 ± 0.33 and 60.99 ± 0.15, respectively. Besides, the ENC values of individual genes of different lineages exhibited distinguishing characteristics, especially the AF1 lineage, whose ENC values were much far away from the rest of the f lineages in almost every gene (Figure 2B). Significant differences (adjusted *p* < 0.05) were discovered in the average ENC values of the ten genes and different lineages of each gene (Supplementary Figure S2). These results suggested a low but lineage-specific CUB among the USUV coding sequences.

### USUVs have evolved into lineage-specific RSCU patterns

RSCU analysis was used to explore the patterns of and preferences for codon usage among genes. Here we found that except for Phe without CUB, all the remaining 17 amino acids had preferred codons (RSCU >1.0; Supplementary Table S3). Specifically, 29 of 59 synonymous codons were classified as preferred codons, eighteen of them are G/C-ended (12 C-ended; 6 G-ended) and eleven were A/U-ended (7 A-ended; 4 U-ended). This means C-and A-ended codons are preferred in the USUV. Among the preferred codons, three codons (AGA, CUG, GGA) were over-represented (RSCU >1.6). Similarly, nine codons (UUA, GUA, UCG, CCG, ACG, GCG, CGA, CGC, CGU) were under-represented (RSCU <0.6).

**Figure 3:**
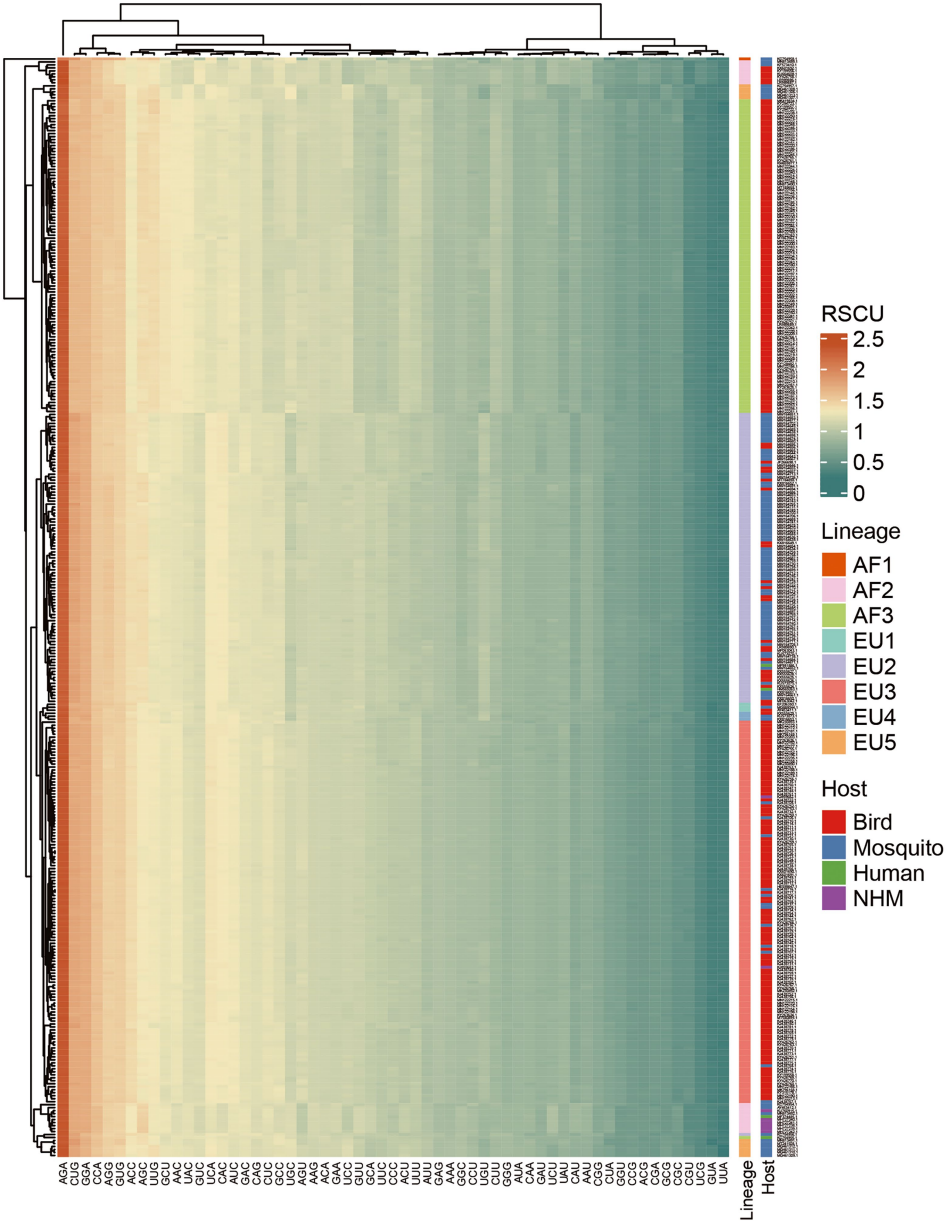
Heatmap of mean Relative synonymous codon usage (RSCU) values among the 368 complete coding sequences of USUV. Each row represents a USUV isolate and each column represents a codon. Rows and columns are clustered.

Taking lineage information into consideration, we found that preferred codons varied. A total of 35 codons were preferred by at least one lineage, while only 21 of them were preferred by all eight lineages. The preferred codons of some amino acids were different among the lineages (Supplementary Table S3). Moreover, unlike the other lineages, the AF1 lineage had five over-represented codons and three of hem are unique (GUG, CCA, and AGG). The underrepresentation analysis result was more complex. A total of 11 codons were under-represented in at least one lineage, and 6 of them were under-represented in all eight lineages. The heatmap also indicated distinctively lineage-specific RSCU patterns (Figure 3). The lineage-specific codon usage patterns underscore the independent evolutionary history of USUV strains. Additionally, we found that the common preferred codons (RSCU >1.0) and unpreferred codons (RSCU <1.0) were neither completely harmonious nor opposite in USUV compared to any of the hosts (Supplementary Table S3).

### Trends of codon usage variations in USUV

**Figure 4:**
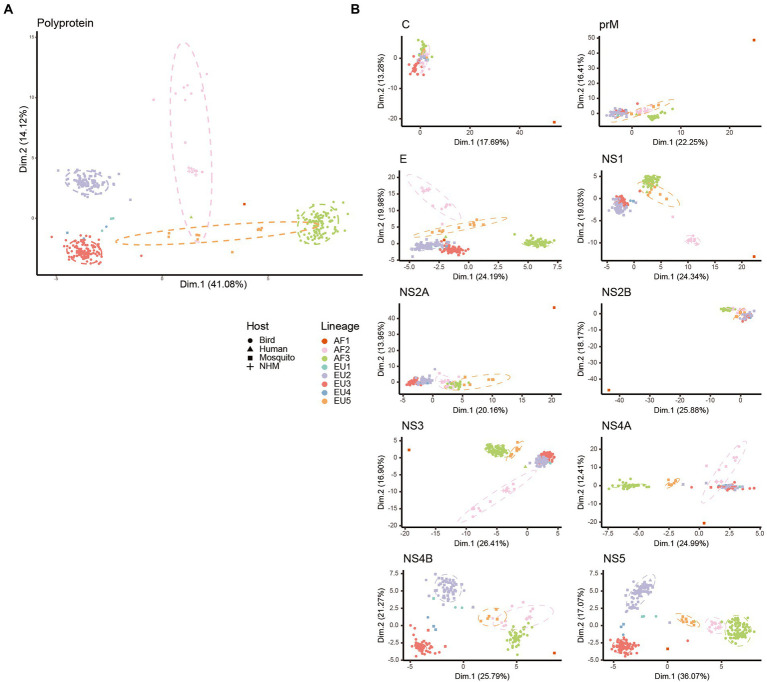
Principal component analysis (PCA) biplot diagram showing the trends of codon usage variation in USUV. PCA biplots were performed on the RSCU values of whole coding sequences **(A)** and every gene **(B)**. Every point represents a USUV strain. The point shape and color are depicted according to isolation host and lineage classification. The ellipses are drawn in a 95% confidence interval.

PCA analysis was performed to explore the synonymous codon usage variations among the USUV isolates. The first and second axes accounted for 41.08% and 14.12% of the total codon usage variation (Figure 4A). The strains were mainly grouped into five well-defined clusters, corresponding to 5 of 8 lineages (AF2, AF3, EU2, EU3, and EU5). The remaining three lineages were scattered probably due to their small population size. Specifically, the AF2, AF3, EU2, and EU3 lineages were grouped into distinctly separate clusters. However, the 95% confidence ellipse of the EU5 lineage had a few overlapping with that of the AF2 and AF3 lineages. The AF1 did not closely group with any clusters/lineages. In addition, we also performed PCA of individual genes based on lineages and hosts (Figure 4B). The unique codon usage of the AF1 lineage is retained in all genes. Instead, the distinctly separated clusters of the five lineages were kept in some genes such as *E* and *NS5*, whereas much more overlapping tendencies were found in the other genes such as *C* and *NS2B*. All above, these results reconfirm a lineage-specific codon usage of USUV and suggest a common ancestry, but the independent and varied divergence history at the levels of individual genes.

### Relative dinucleotide abundances influence the codon usage pattern of USUV

**Figure 5:**
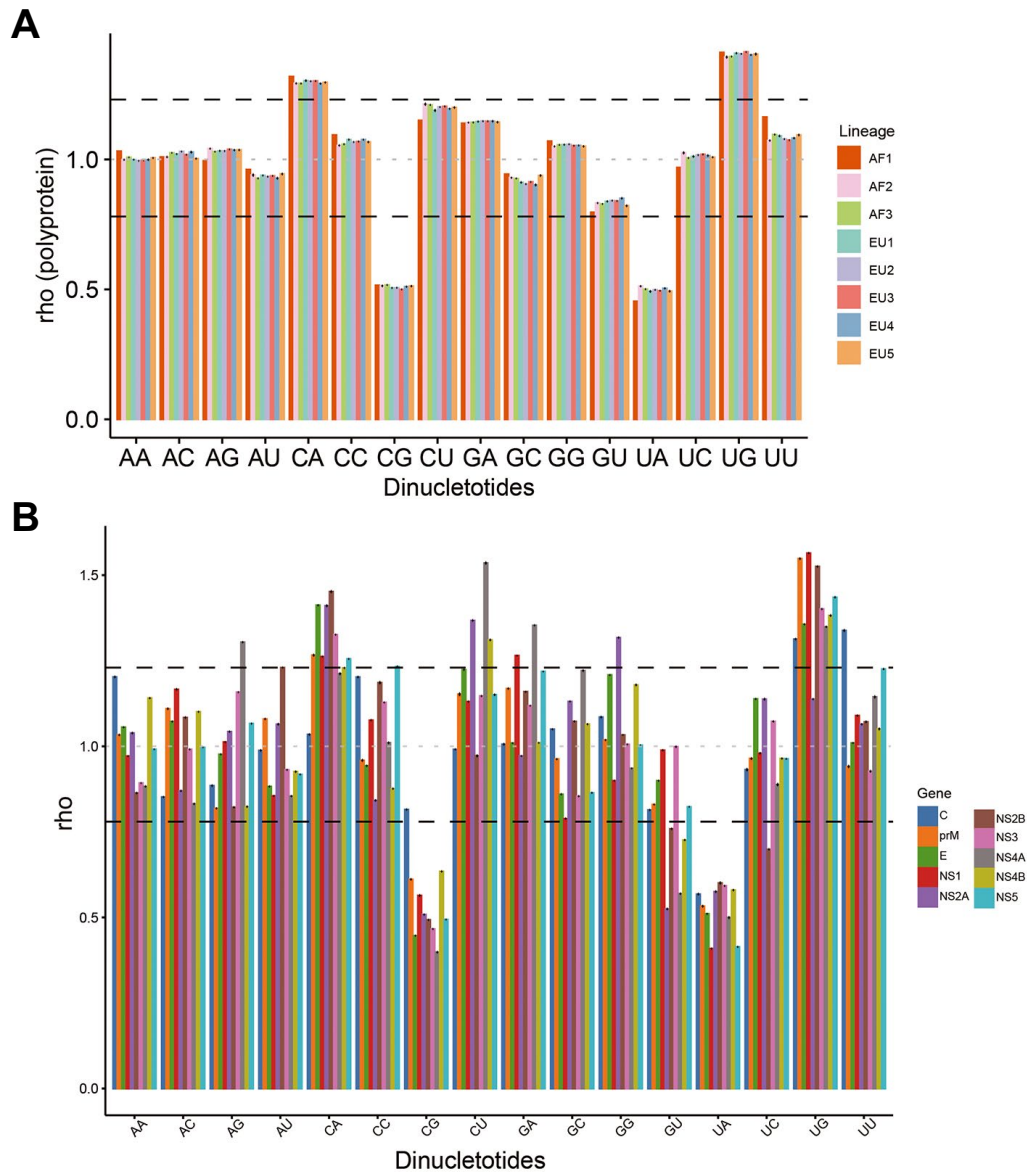
The relative dinucleotide abundances of USUV coding sequences. **(A)** Whole coding sequences. The different colors represent lineage classification. **(B)** The different colors represent different genes. The black dash lines indicate 1.23 and 0.78, respectively. The grey line indicates the expected frequency (1.0).

To determine the potential effect of dinucleotide frequencies on codon usage, the relative abundances of the 16 dinucleotides were calculated. The relative abundance of dinucleotides was found to be not random. Dinucleotides CpA and UpG were over-represented (*rho* ≥ 1.23), whereas dinucleotides CpG and UpA were under-represented (*rho* ≤ 0.78) in the complete coding sequences of the eight USUV lineages (Figure 5A). Dinucleotides CpU was marginally over-represented, especially in the AF2-3 lineages. Meanwhile, the relative abundance of every dinucleotide varied among different lineages. For example, the *rho* of dinucleotide UpC was less than 1 in AF1 but greater than 1 in the other lineages. These results indicated that eight lineages of USUV had a unique and biased dinucleotide usage pattern.

To further test the effect of dinucleotide usage on codon usage bias, we compared the over-and under-represented dinucleotides with the preferred and under-represented codons. Among the thirteen CpA-or UpG-containing codons, seven codons (CUG, UUG, GUG, UCA, CCA, ACA, and CAG) were preferred in all the lineages, three codons (GCA, CAC, and UGC) were preferred in at least six lineages, two codons (CAU and UGU) were preferred in the AF1 and EU4 lineage, respectively, only single codon (CAA) was not preferred (RSCU ≤0.86; Supplementary Table S3). Similarly, among the fourteen CpG-or UpA-containing codons, six codons (UUA, GUA, UCG, CGA, CGC, and CGU) were under-represented in all the lineages, two codons (ACG and GCG) were under-represented in 7 of 8 lineages, one codon (CCG) was under-represented in the five lineages (AF2, EU1, and EU3-5), one codon (CUA) was under-represented in AF1 lineage alone, and three codons (AUA, UAU, and CGG) was not under-represented but also not preferred (RSCU 0.69–084) in any lineage. Interestingly, one codon (UAC [Tyr]) containing under-represented dinucleotide UpA was preferred in all eight lineages. These data showed that biased dinucleotide abundance influenced the codon usages of the eight lineages of USUV.

Additionally, we analyzed the relative dinucleotide abundance at individual genes level. A diverse dinucleotide usage landscape was observed (Figure 5B; Supplementary Figure S3). Dinucleotide CpA was not over-represented in the *C* genes of all lineages (RSCU 1.00–1.07) and *NS1*, *NS4A*, and *NS4B* genes of several lineages. Dinucleotide UpG was not over-represented in the *C* (AF1) and *NS2A* (except AF1) genes. In contrast, dinucleotide ApG in the *NS4A* (except AF1), GpA in the *NS1* and *NS4A*, GpG in the *NS2A* (except AF1), and UpU in the *C* gene of all lineages were over-represented, respectively. Meanwhile, dinucleotide CpG was not under-represented in the *C* gene of all lineages except the AF2, whereas dinucleotide GpU was under-represented in the *NS2A*, *NS2B*, *NS4A,* and *NS4B* genes of all eight lineages. Dinucleotide UpC was under-represented in the *NS2B* gene of all lineages except AF1, too. These results are consistent with distinct ENC values and codon usage patterns among the different genes and lineages, indicating that dinucleotide frequencies probably have an influence on the codon usage of USUV.

### Both natural selection and mutation pressure shape the codon usage pattern of USUV

**Figure 6:**
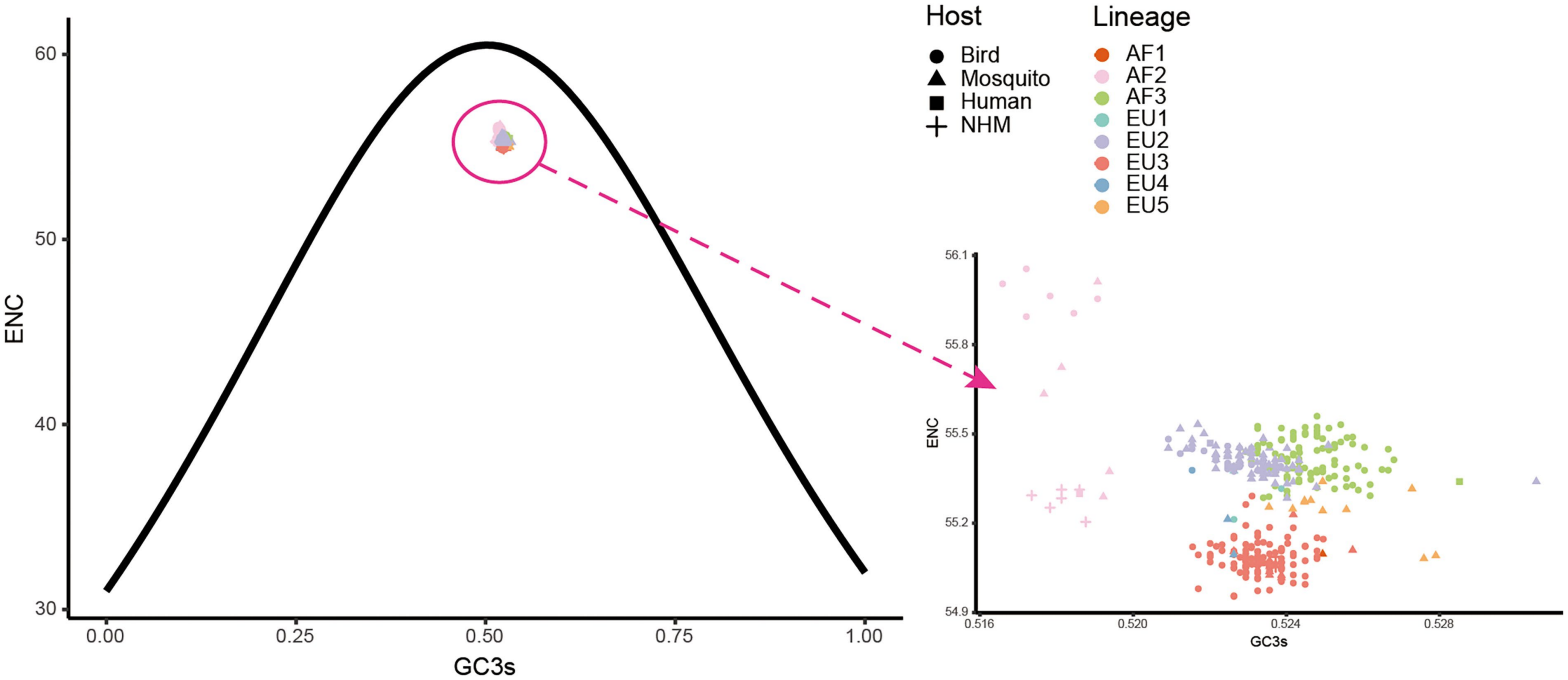
The effective number of codons (ENC) plot of whole coding sequences of USUVs. The solid curve represents the expected ENC values when the codon usage was only influenced by the GC3s composition. The point shape and color are depicted according to isolation host and lineage classification.

To determine the factors that influence the codon usage pattern, ENC plots and correlation analyses were performed. In the ENC-GC3s plot of complete coding sequences, all USUV isolates were lying below the expected ENC curve (Figure 6). The strains from AF2, EU3, AF3, EU2, and EU5 formed five distinguishing clusters, albeit clusters of the later three lineages had a few overlaps. This indicated natural selection dominated the codon usage of all USUV strains. However, ENC plots of individual genes showed that the effects of natural selection and mutation pressure on codon usage varied. For example, all NS2B and NS4B coding sequences lay above the expected ENC curve, except for the AF1, showing the dominant role of mutation pressure in these genes (Supplementary Figure S4). These results suggested that both mutation and natural selection shape the codon usage patterns of USUVs.

Furthermore, correlation analysis revealed a mixture of significant (*p* < 0.05) and non-significant correlations between nucleotide compositions and codon compositions (Supplementary Figure S5). Especially, the first two axes of PCA had significant correlations with almost all the indices, including mononucleotides, A3s, C3s, G3s, U3s, GC1s, Aromo, and ENC. A remarkable relationship between mononucleotides, A3s, C3s, G3s, U3s, and ENC was observed as well (all |*r*| ≥ 0.63). These results reconfirmed the combined role of mutation pressure and natural selection in the codon usage propensities of USUV.

### Natural selection is the major driver of USUV codon usage

**Figure 7:**
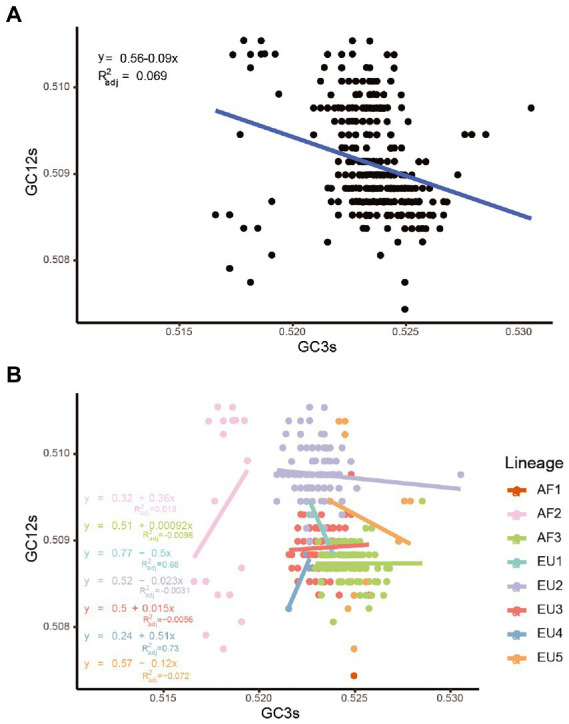
Neutrality analysis of the USUV whole coding sequences for all strains **(A)** and different lineages **(B)**.

Once we recognized that both natural selection and mutation pressure contributed to the CUB of the USUV, a neutrality analysis was conducted to determine the magnitude of the two forces. Regarding complete coding sequences, neutrality analysis showed a low but significant correlation between GC12s and GC3s values among all the strains (*R*^2^_adj_ = 0.069, *p* < 0.0001). The slope of the regression line was inferred to be-0.09, according to which mutation pressure (relative neutrality) was 9% and natural selection (the relative constraint on GC3s) was 91% (Figure 7A), indicating the principal effect of natural selection on the codon usage of USUV. Based on individual lineages analyses, the slopes of linear regression were 0.36, 0.00092, −0.5, −0.023, 0.015, 0.51, and −0.12 for the AF2-3 and EU1-5 lineages, respectively (Figure 7B). Therefore, the mutation pressure accounted for 36%, 0.092%, 50%, 2.3%, 1.5%, 51%, and 12%, whereas natural selection accounted for 64%, 99.908%, 50%, 97.7%, 98.5%, 49% and 88% in the corresponding lineages, respectively. The AF1 lineage had no linear regression result due to its single population size. Although mutation pressure explained 50% and 51% in the EU1 and EU4 lineage, respectively, all the correlations were not statistically significant in the seven lineages (*p* > 0.2392). These results reaffirmed the dominant influence of natural selection.

In addition, we performed the neutrality analysis in 10 genes similarly. We found that despite significant correlations between GC12s and GC3s were observed in all genes except the *C* and *NS5*, with relative neutrality ranging from 1.3% (*NS5*) to 23% (*NS1*), mutation pressure was the minor force in all genes, irrespective of lineages (Supplementary Figure S6A). Taking the lineage information into consideration, all absolute values of regression slopes were less than 0.5 and most of them were close to zero or negative (Supplementary Figure S6B). The only exception is the *E* genes of the EU4 lineage, but the coefficient between GC12s and GC3s was-0.5 (*p* = 0.55). In a word, although the different degrees of mutation pressure influence on distinct lineages and individual genes, natural selection predominated the evolution of codon usages in USUV.

### Host-specific codon adaptation patterns in USUV

**Figure 8:**
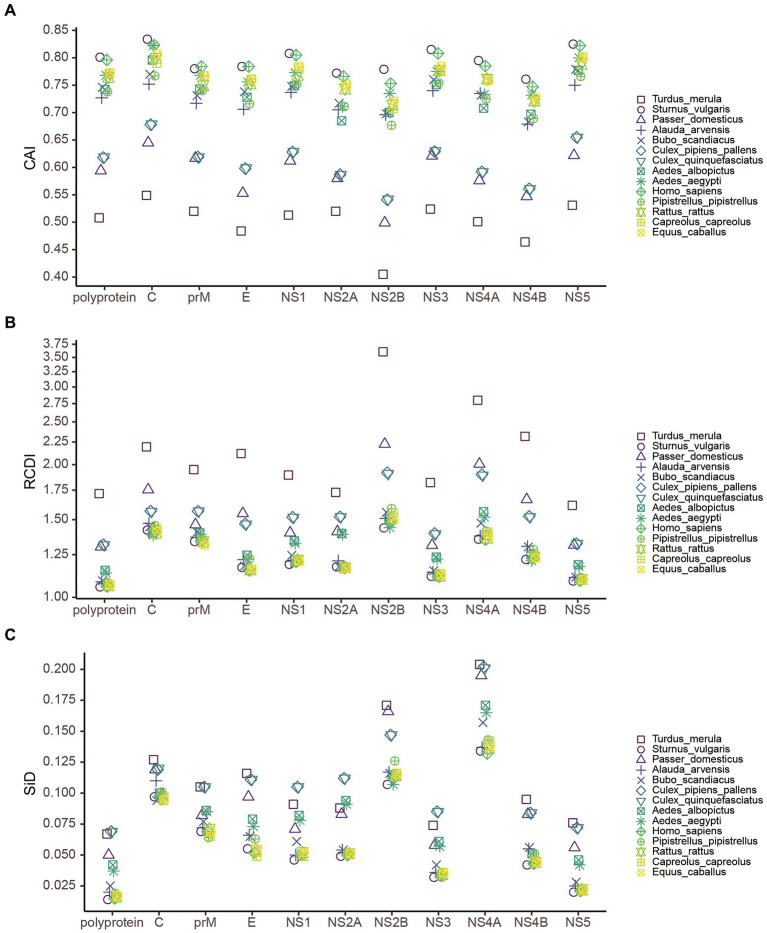
The measure of overall USUV adaption to various potential hosts. **(A)** Codon adaption index (CAI), **(B)** Relative codon deoptimization index (RCDI), and **(C)** The similarity index (SiD) analysis of the codon usage between USUV coding sequences and their hosts. Different reference hosts are depicted in distinct shapes and colors.

**Figure 9:**
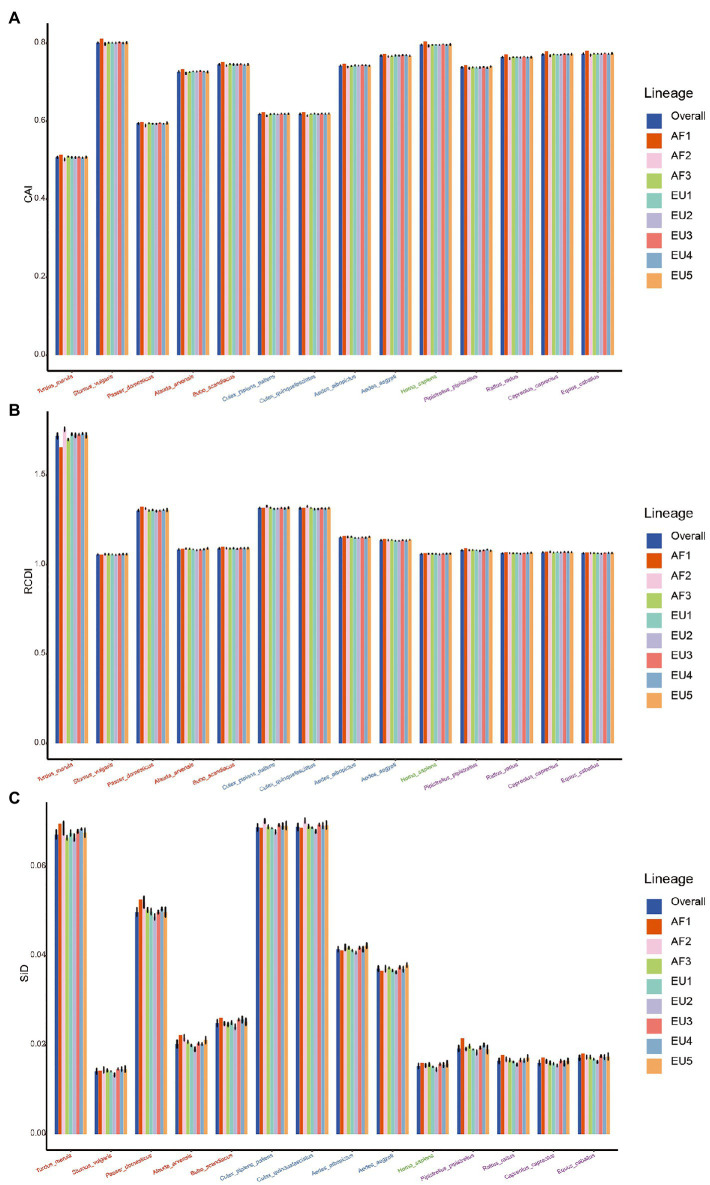
The measure of adaption of distinct lineages of USUV to various hosts. **(A)** CAI, **(B)** RCDI, and **(C)** SiD analysis of the codon usage between the complete coding sequences of the USUV and its hosts. Trends in overall and different lineages are depicted in distinct colors. Error bars represent the standard error of the mean values.

To estimate the relative adaptation of USUV to their hosts and vectors, we performed a CAI analysis. Here we found that the CAI values varied from host to host (Figure 8A). Regarding the whole coding sequence in USUV, the highest CAI values were found in *S. vulgaris* (0.801 ± 0.002), followed by *H. sapiens* (0.796 ± 0.001) and *E. caballuss* (0.773 ± 0.001). The USUV also displayed high CAI values toward the other three NHM hosts. The lowest CAI values were found in *T. merula* (0.508 ± 0.002), followed by *P. domesticus* (0.594 ± 0.002) and *Cx. pipiens* pallens (0.618 ± 0.001). Except for the pair of two *Culex* species, the CAI values of USUV showed statistical significance in all host pairs (adjusted *p* < 0.05). Taking virus lineages into consideration, we observed that the CAI values of different lineages to the same host varied but a similar pattern was still preserved (Figure 9A). In addition, the CAI values for different hosts varied but relatively conserved patterns were maintained at individual genes across different lineages (Figure 8A).

### USUV displays the highest codon deoptimization to *Turdus merula*

To determine the codon usage deoptimization of the USUV coding sequences with their potential hosts, the RCDI values were inferred. The highest three mean RCDI values were obtained relative to *T. merula* (1.719 ± 0.016), *Cx. pipiens* pallens (1.317 ± 0.004), and *Cx. quinquefasciatus* (1.315 ± 0.004), whereas the lowest three RCDI values were obtained relative to *S. vulgaris* (1.057 ± 0.002), *H. sapiens* (1.060 ± 0.002), and *E. caballuss* (1.064 ± 0.002; Figure 8B). Despite the variation, a similar RCDI values pattern of complete coding sequences in USUV was maintained across all lineages (Figure 9B).

### *Cx. quinquefasciatus* plays a significantly stronger selection pressure on USUV

To investigate the potential impact of these hosts on the evolution of codon usage patterns of the USUV, the SiD analysis was performed. The results showed that the overall mean SiD value was highest in *Cx. quinquefasciatus* (0.0689 ± 0.0008) versus the complete coding sequences of USUV (Figure 8C). A slightly smaller but comparable (adjusted *p* = 0.77) SiD value was observed in *Cx. pipiens* pallens (0.0688 ± 0.0008). The SiD values in these two hosts were remarkably larger than that in the other hosts simultaneously (adjusted *p* < 0.0001). When considering the lineage classification of the polyprotein sequences, a similar trend remained in all lineages except for the AF1 (Figure 9C), where the highest SiD value was observed in *T. merula*, indicating that *Cx. quinquefasciatus* played the strongest influence on the USUV codon usage choices in most of the lineages.

Additionally, the SiD analysis was performed on ten genes of the eight lineages. The mean SiD value for *T. merula* was found to be highest in the *C*, *E*, *NS2B*, *NS4A*, *NS4B*, and *NS5* genes, while that was found for *Cx. quinquefasciatus* in the *prM*, *NS1*, *NS2A*, and *NS3* genes, without consideration for lineages (Figure 8C). There is no significant difference between the SiD values for *Cx. quinquefasciatus* and *Cx. pipiens* pallens in all individual genes. In summary, *Cx. quinquefasciatus* and *T. merula* exerted larger selection pressure on the various genes of different lineages.

## Discussion

In this study, we conducted a comprehensive analysis of the phylogenetic relationships and the codon usage patterns of the USUVs to understand their molecular evolution. Our phylogenetic tree divided the USUV strains into eight lineages. This result is consistent with the previous reports (Cadar et al., 2017; Vilibic-Cavlek et al., 2020; Zecchin et al., 2021). The PCA analysis confirmed the outcome of phylogenetic analysis, as the well-defined clusters corresponded to the phylogenetic lineages. This also indicates the USUV has evolved into lineage-specific RSCU patterns, which implies a non-negligible role of evolutionary processes affecting its codon usage.

The genomic composition can greatly affect the CUB (Nasrullah et al., 2015). Our data showed that G and A were more abundant in USUV coding sequences. Besides, the RSCU analysis showed that C-end and A-end codons were mostly preferred, which suggested that protein-coding sequences of USUV were inclined to use C/A-ended codons and the nucleotide composition changes derived from distinct factors may play a pivotal role in its CUB. The C and A-ended codons may help the genes of USUV to be translated more accurately (Sun and Zhang, 2022). A similar phenomenon was also observed in other RNA viruses such as Zika virus (ZIKV; Butt et al., 2016) and Chikungunya virus (CHIKV; Butt et al., 2014). ENC analysis showed that the overall mean ENC value of all USUV isolates was 55.30, indicating a slightly biased and conserved codon usage. Similar low CUB has also been found in many RNA viruses, such as ZIKV (53.93; Butt et al., 2016), JEV (55.30; Singh et al., 2016), WNV (53.81), Ebola virus (EBOV; 55.57; Luo et al., 2020), Alongshan virus (ALSV; 55.07; Sura et al., 2021), and Marburg virus (MARV; ENC, 54.2; Nasrullah et al., 2015). Previous studies suggested lower CUB could reduce the translation resources competition between viruses and their host, which improves viral replication efficiency (Nasrullah et al., 2015; Butt et al., 2016). Therefore, it seems that the low CUB of USUV may have prompted maintaining its circulation in various hosts with different codon usage preferences.

Dinucleotide abundance could affect the codon usage bias, which has been reported in many viruses(Kariin and Burge, 1995; Cheng et al., 2013). Here we found a unique and non-random dinucleotide pattern in USUV. More specifically, dinucleotide CpG and UpA were under-represented while the CpA and UpG were over-represented. Similar dinucleotide representation was also observed in WNV (Moratorio et al., 2013) and JEV (Singh et al., 2016). It has been postulated that the CpG depletion is associated with the immunostimulatory properties of unmethylated CpG, which is recognized by the host’s innate immune system as a pathogen signature (Shackelton et al., 2006). As result, vertebrates have an avoidance of dinucleotide CpG in their genomes (Parisien et al., 2013; Takata et al., 2017) and CpG is suppressed in genomes of many vertebrate RNA viruses, especially the exclusive repression in vertebrate-infecting *Flavivirus* group (Lobo et al., 2009). Besides, most vertebrates possess a latent intracellular interferon-induced ribonuclease (RNase L), which has a preference for the degradation of UpA-rich RNA and activates apoptotic pathways (Shaw and Kamen, 1986; Beutler et al., 1989). Therefore, the under-representation of CpG and UpA might allow the USUV to more effectively replication and transmission among vertebrate hosts, and the over-representation of CpA and UpG may be the result of ADAR editing or compensating for the deficiency of CpG and UpA (Talia and Adi, 2020). Moreover, these results suggest that dinucleotide abundance has affected the codon usage of USUV, but the overall impact is relatively limited because of the exception among the dinucleotide abundances and codon preferences in USUV genomes.

To clarify the factors that influenced the codon usage patterns of USUV, we performed a detailed ENC-GC3s plot, correlation analysis, and neutrality analysis. When the ENC and GC3s values of complete coding sequences of USUV were depicted, we found that all strains were lying below the expected ENC curve, demonstrating that natural selection overall predominated the codon usage of USUV over mutation selection. However, a few contrary phenomena were observed when this analysis was conducted at the level of the individual gene, showing that the effect of mutation pressure was not entirely lacking, especially in some genes such as the *NS2B* and *NS4B*. Correlation analysis reaffirmed the role of natural selection and mutation pressure. Moreover, detailed neutrality analyses demonstrated the dominant role of natural selection in shaping the CUB of USUV, regardless of the lineages and genes. Our results are consistent with the other viruses in the genus *Flavivirus*, such as ZIKV (Butt et al., 2016) and JEV (Singh et al., 2016).

The codon usage pattern of the virus is likely affected by its host. Here, we found a mixture of coincidence and antagonism in the codon usage between the USUV and its hosts. This pattern indicated that multiple hosts may have applied selection pressure on the codon usage of the USUV, like ZIKV (Butt et al., 2016) but different from MARV (Nasrullah et al., 2015). Moreover, the CAI and RCDI analysis revealed a disproportionate level of adaptation to its different hosts and vectors, indicating that natural selection exerted pressure on the codon usage of USUVs, although at the variable level from varied hosts. The high adaptation to *H. sapiens* and other mammals suggested the USUV has adjusted its codon usage choice to employ the translation resources more efficiently in mammals, warning of the potential role of these animals in USUV amplification and epidemic. Low adaptation to *T. merula* and *Cx. pipiens* indicated that USUV has maintained a relatively low translation rate of viral proteins in these hosts, which may be milder harmful to these hosts but supports stable survival and spread of progeny viruses. The lowest adaptation to *T. merula* also suggested that it is the most probable primary natural reservoir of USUVs, which is in line with previous reports (Renke et al., 2017; Roesch et al., 2019). However, our findings are partly inconsistent with Zecchin et al. (2021), who observed lower CAI values for *S. vulgaris* than *H. sapiens*. This discrepancy might be owing to the different codon usage of *S. vulgaris* used. Yet further investigation is necessary. In addition, as revealed by the SiD analysis, *Cx. quinquefasciatus* have exerted larger selection pressure on the codon usage of 7 of 8 USUV lineages, implying that *Cx. quinquefasciatus* is a potential new favored vector of USUV. When evaluated in individual genes, the most selection pressure of codon usage of USUV came from *T. merula* and *Cx. quinquefasciatus*, depending on the genes. Accordingly, it makes sense that USUV evolved a lower level of adaptation with its natural reservoir and primary vector than the terminal hosts to facilitate their long-term survival and circulation, as observed in MARV (Nasrullah et al., 2015) and EBOV (Luo et al., 2020).

## Conclusion

In conclusion, this study reveals a slightly biased and lineage-specific codon usage pattern within USUVs. Mutational pressure, natural selection, dinucleotide abundance, and evolutionary processes collectively shaped the codon usage of USUVs. Specifically, natural selection predominated over the other factors. In addition, we found that USUVs have evolved a host-specific adaptation to various hosts and vectors, especially a high fitness to mammals, including humans. The findings of this study improve our insights into the evolution of USUVs that will consolidate future USUV research. Moreover, our results suggest that further epidemiologic monitoring and pathogenicity studies in these high-fitness hosts are particularly required to confront the potential risk of cross-species transmission and outbreak.

## Data availability statement

The original contributions presented in the study are included in the article/Supplementary material, further inquiries can be directed to the corresponding author.

## Author contributions

JZ and SW conceptualized and designed the study. JZ collected data, performed the analyses, and drafted the manuscript. YX and ZZ revised the manuscript and participated in the interpretation of the result. SW supervised the study and revised the manuscript. All authors contributed to the article and approved the submitted version.

## Funding

This work was supported by the National Natural Science Foundation of China (https://www.nsfc.gov.cn/) under grant (numbers 81830101 and 82072285); and Ministry of Science and Technology of China under grant (number 2018YFA0902300).

## Conflict of interest

The authors declare that the research was conducted in the absence of any commercial or financial relationships that could be construed as a potential conflict of interest.

## Publisher’s note

All claims expressed in this article are solely those of the authors and do not necessarily represent those of their affiliated organizations, or those of the publisher, the editors and the reviewers. Any product that may be evaluated in this article, or claim that may be made by its manufacturer, is not guaranteed or endorsed by the publisher.

## Footnotes

^1^ http://codonw.sourceforge.net/culong.html#CodonW

## Supplementary material

SUPPLEMENTARY FIGURE 1 Boxplots of the GC, GC percentage at the first (GC1s), second (GC2s), third (GC3s) codon position values of USUV strain in various lineages **(A)**, isolation hosts **(B)**, and different genes **(C)**. Every point represents a strain. All differences with *P* < 0.01 are indicated. ^**^*P* < 0.001; ^***^*P* < 0.0001; using two-sided unpaired Wilcoxon rank test (BH-corrected).

SUPPLEMENTARY FIGURE 2 The effective number of codons (ENC) values distribution. **(A)** The overall ENC values comparison among the different genes. **(B–K)** show the ENC values of various lineages of the ten genes, respectively. All differences with *P* < 0.01 are indicated. ^**^*P* < 0.001; ^***^*P* < 0.0001; using two-sided Dunn’s test (BH-corrected).

SUPPLEMENTARY FIGURE 3 The relative abundance of 16 dinucleotides in ten genes. Different colors represent lineage classification. The black dash lines indicate 1.23 and 0.78, respectively. The grey line indicates the expected frequency (1.0).

SUPPLEMENTARY FIGURE 4 The effective number of codons (ENC) plots of different genes of the 368 USUV strains. The solid curve represents the expected ENC values when the codon usage was only influenced by the GC3s composition. The point shape and color are depicted according to isolation host and lineage classification.

SUPPLEMENTARY FIGURE 5 Spearman’s correlation analysis among the nucleotide composition, the effective number of codons (ENC), Aromo, Gravy, and the first two axes of Principal component analysis (PCA) in USUV complete coding sequences. Dark red and blue means positive and negative correlation, respectively. Deeper color darkness means a higher correlation. Non-significant (*P* < 0.05) correlations are not shown.

SUPPLEMENTARY FIGURE 6 Neutrality analysis of the USUV genes for all strains **(A)** and different lineages **(B)**.
