# Supplementary figures and images for: A comprehensive analysis of Usutu virus (USUV) genomes revealed lineage-specific codon usage patterns and host adaptations
Source: Front Microbiol. 2023 Jan 12;13:967999. doi: 10.3389/fmicb.2022.967999 (PMC9878346; doi:10.3389/fmicb.2022.967999)

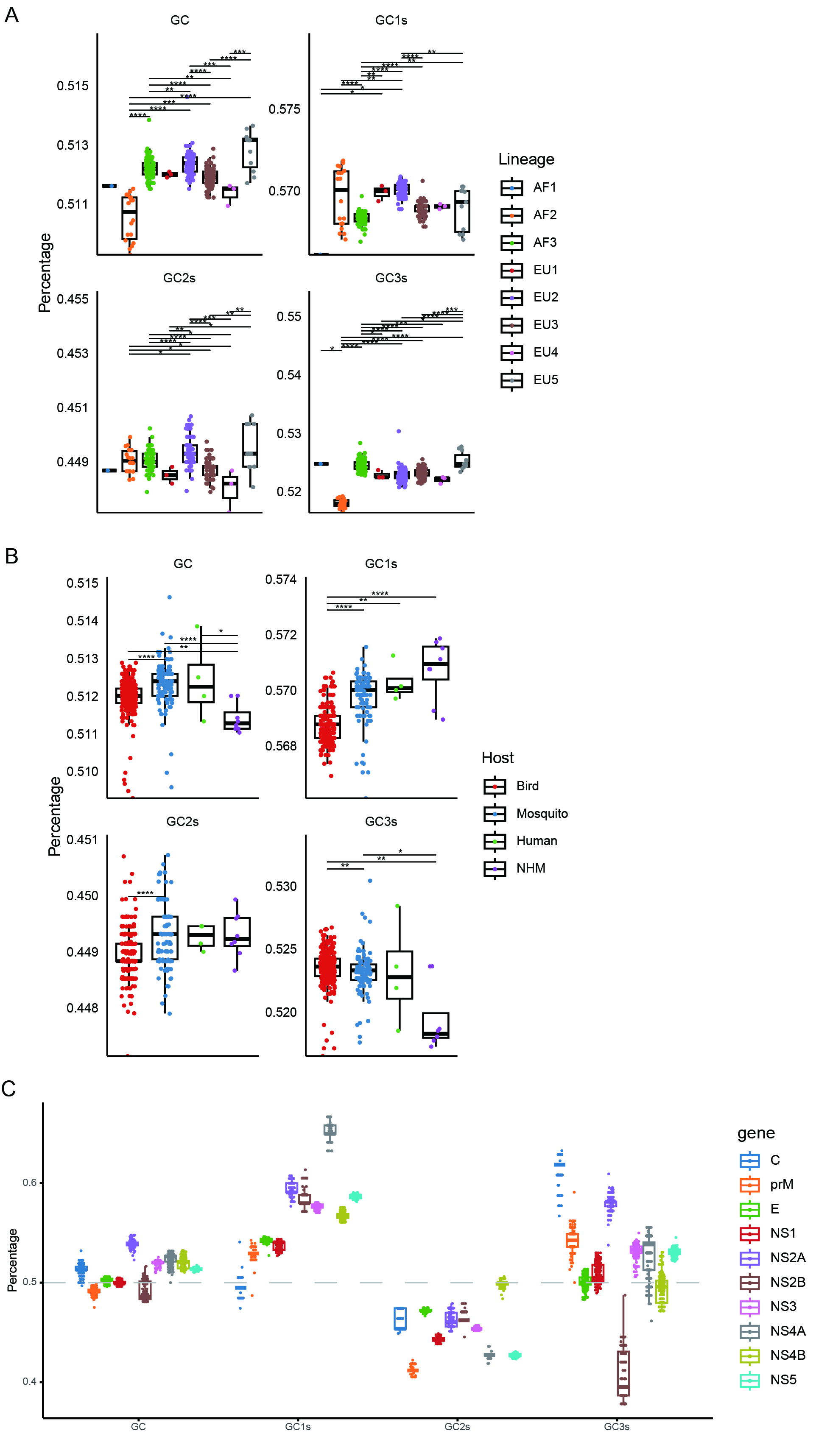

Supplement: SUPPLEMENTARY FIGURE 1 — Boxplots of the GC, GC percentage at the first (GC1s), second (GC2s), third (GC3s) codon position values of USUV strain in various lineages (A), isolation hosts (B), and different genes (C). Every point represents a strain. All differences with P < 0.01 are indicated. **P < 0.001; ***P < 0.0001; using two-sided unpaired Wilcoxon rank test (BH-corrected). [file Image_1.JPEG]

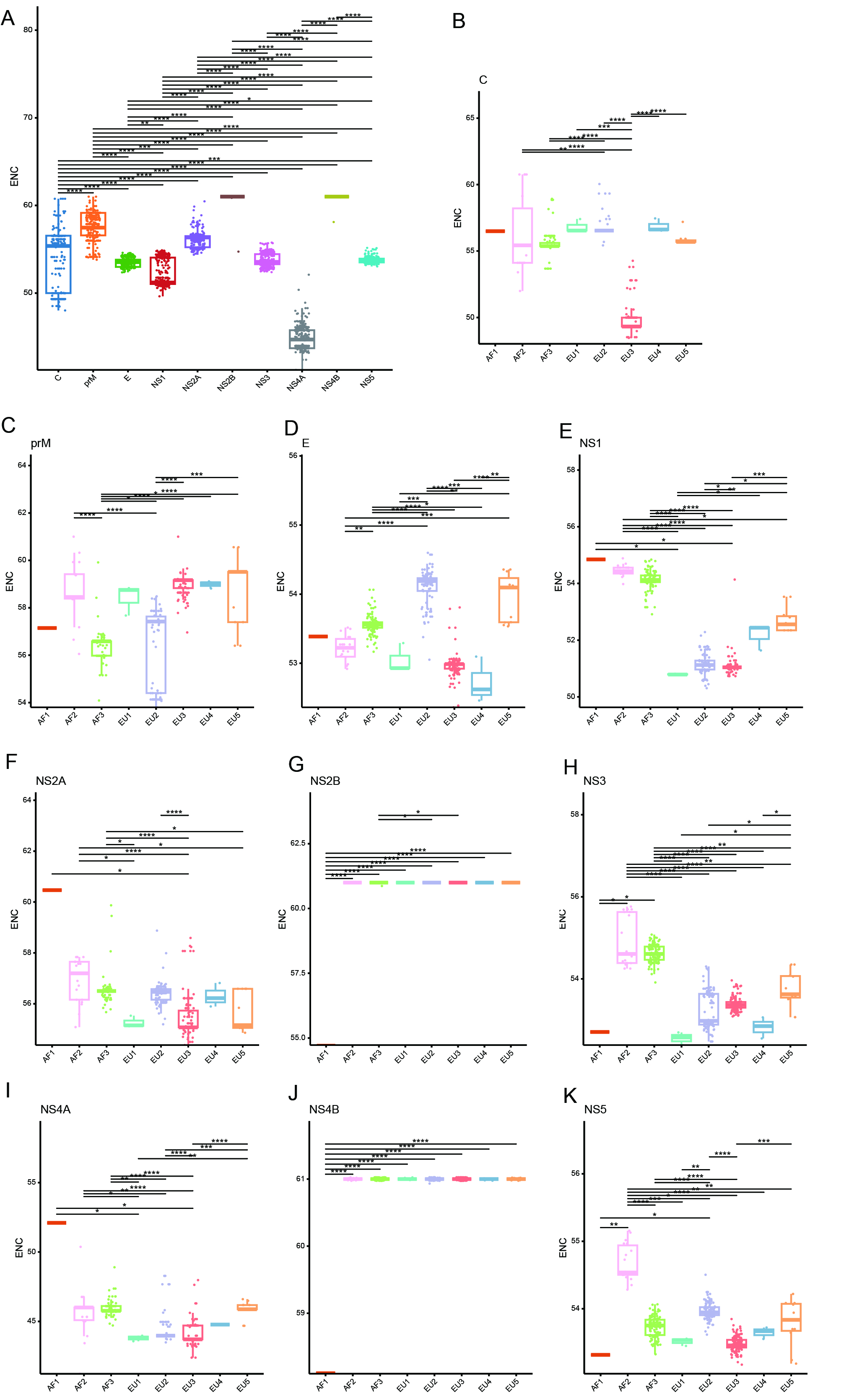

Supplement: SUPPLEMENTARY FIGURE 2 — The effective number of codons (ENC) values distribution. (A) The overall ENC values comparison among the different genes. (B–K) show the ENC values of various lineages of the ten genes, respectively. All differences with P < 0.01 are indicated. **P < 0.001; ***P < 0.0001; using two-sided Dunn’s test (BH-corrected). [file Image_2.JPEG]

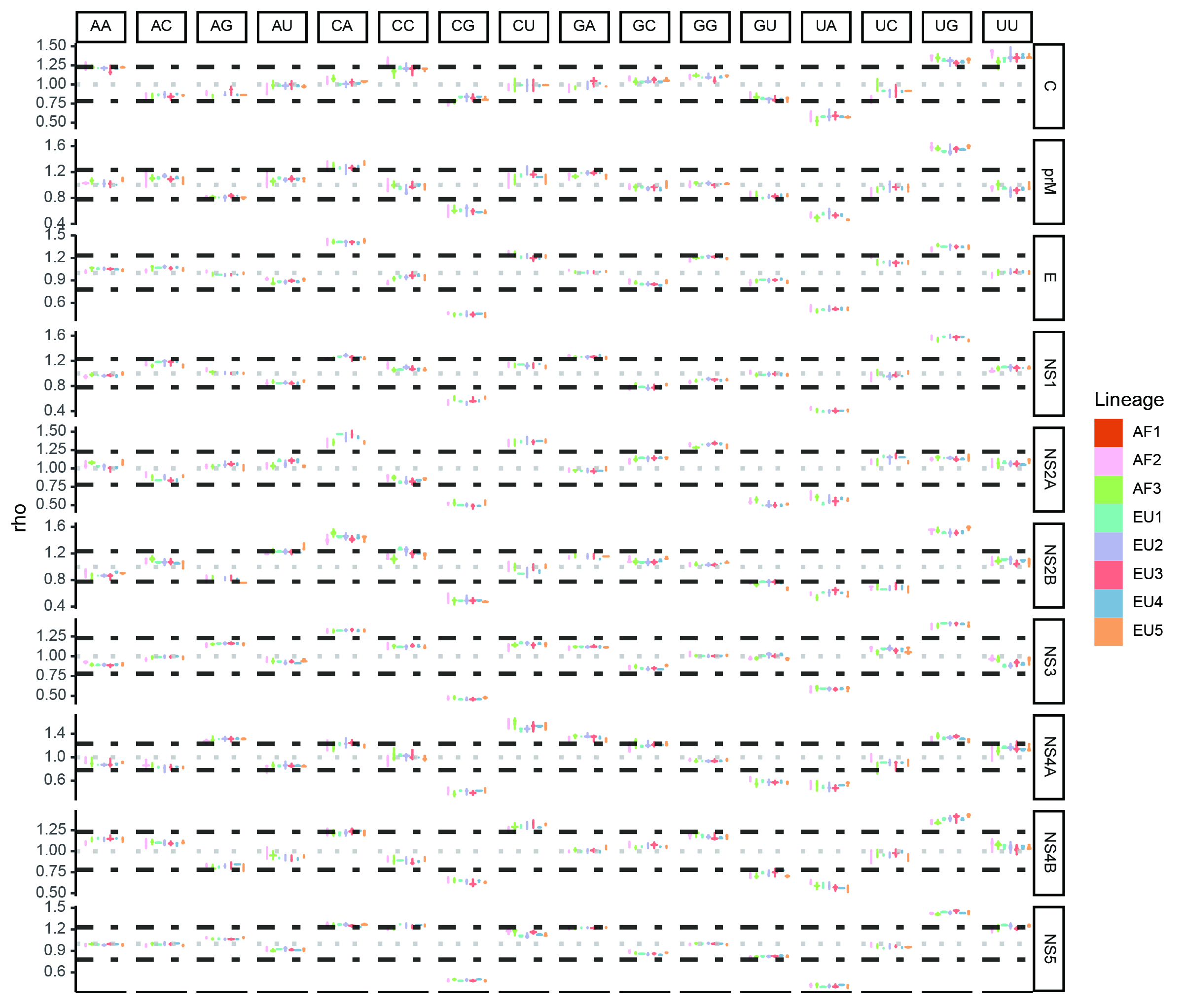

Supplement: SUPPLEMENTARY FIGURE 3 — The relative abundance of 16 dinucleotides in ten genes. Different colors represent lineage classification. The black dash lines indicate 1.23 and 0.78, respectively. The grey line indicates the expected frequency (1.0). [file Image_3.JPEG]

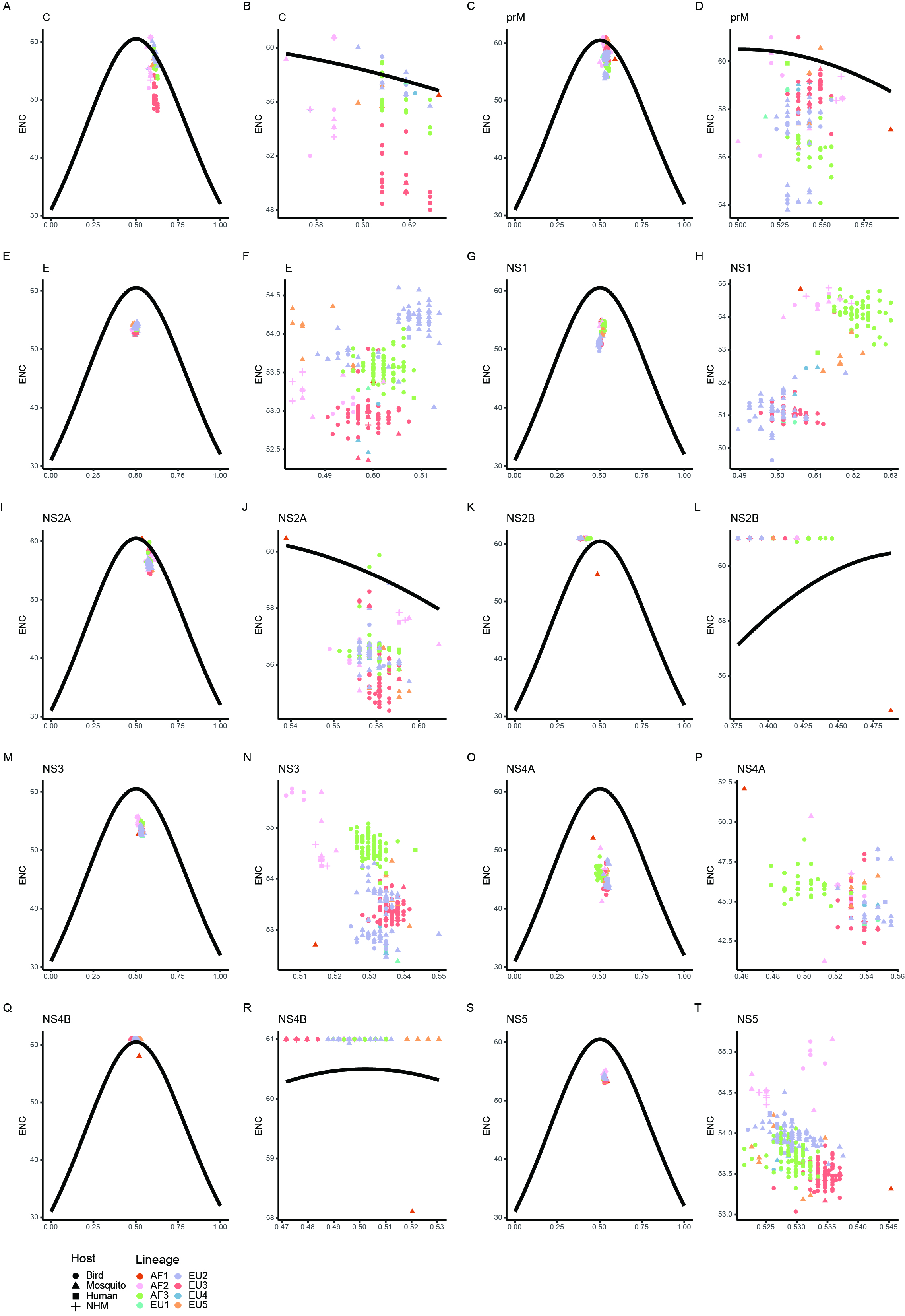

Supplement: SUPPLEMENTARY FIGURE 4 — The effective number of codons (ENC) plots of different genes of the 368 USUV strains. The solid curve represents the expected ENC values when the codon usage was only influenced by the GC3s composition. The point shape and color are depicted according to isolation host and lineage classification. [file Image_4.JPEG]

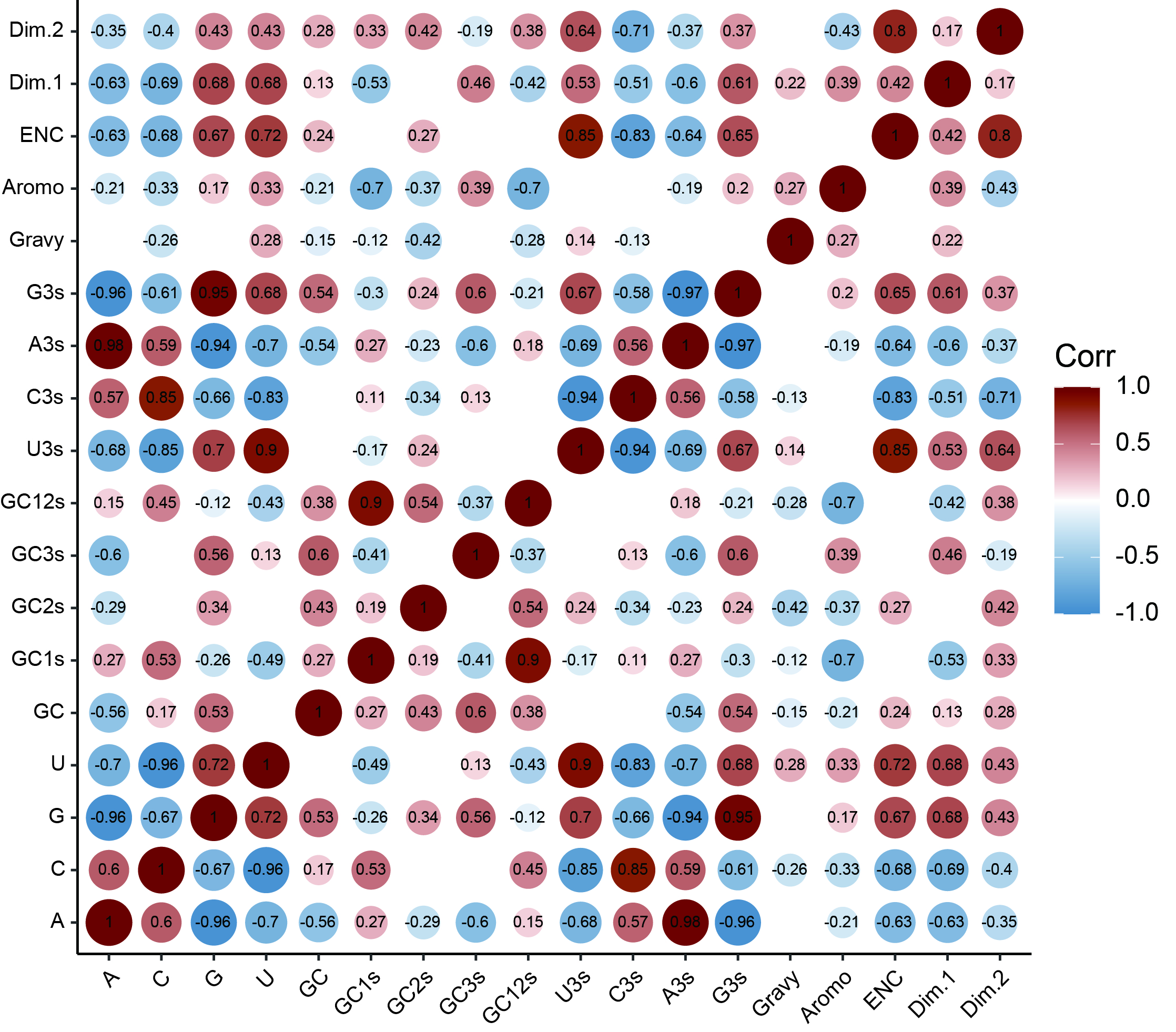

Supplement: SUPPLEMENTARY FIGURE 5 — Spearman’s correlation analysis among the nucleotide composition, the effective number of codons (ENC), Aromo, Gravy, and the first two axes of Principal component analysis (PCA) in USUV complete coding sequences. Dark red and blue means positive and negative correlation, respectively. Deeper color darkness means a higher correlation. Non-significant (P < 0.05) correlations are not shown. [file Image_5.JPEG]

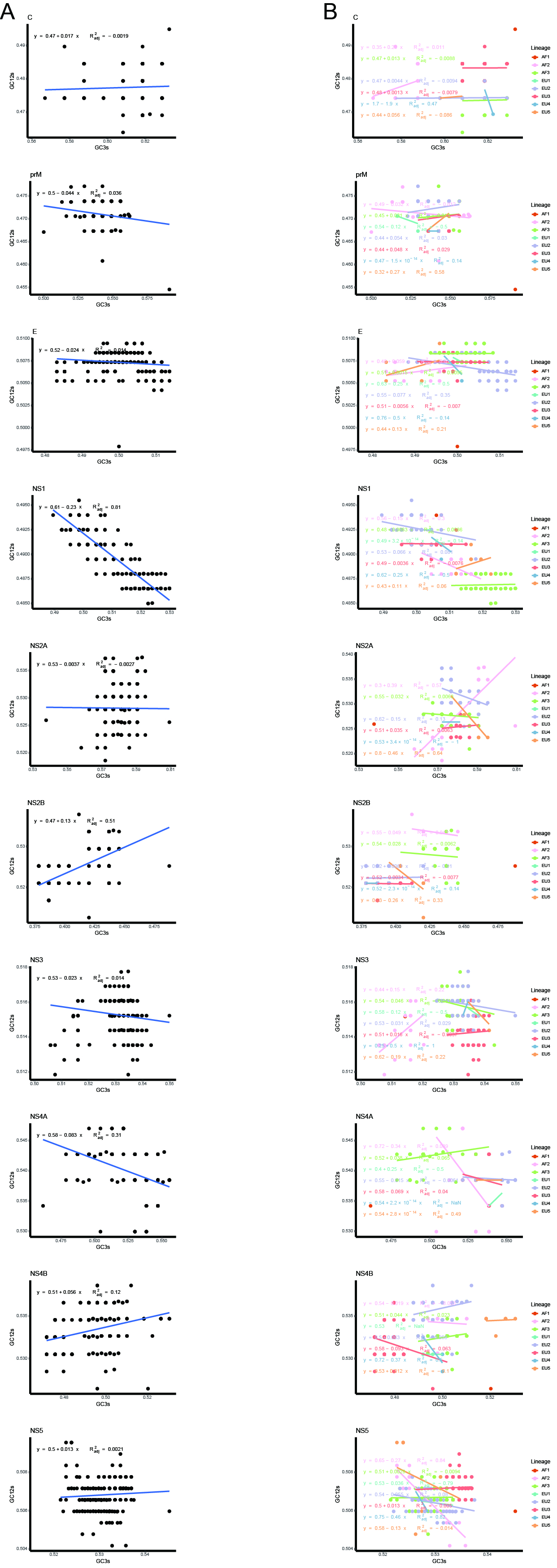

Supplement: SUPPLEMENTARY FIGURE 6 — Neutrality analysis of the USUV genes for all strains (A) and different lineages (B). [file Image_6.JPEG]
